# Hand dysfunction in scleroderma patients

**DOI:** 10.1590/S1516-31802011000500012

**Published:** 2011-09-01

**Authors:** Thelma Larocca Skare, Bruna Leticia Toebe, Carla Boros

**Affiliations:** I MD, PhD. Head of Rheumatology Service, Hospital Universitário Evangélico de Curitiba, Curitiba, Paraná, Brazil.; II MD. Rheumatology Service, Hospital Universitário Evangélico de Curitiba, Curitiba, Paraná, Brazil.; III MD. Rheumatology Service, Hospital Universitário Evangélico de Curitiba, Curitiba, Paraná, Brazil.

To the editor

Scleroderma is a disease characterized by functional and structural abnormalities in blood vessels and fibrous involvement of skin and internal organs.^1^ Although the treatment effort is directed mostly against visceral lesions that can diminish life expectancy, scleroderma patients may also experience difficulties with less serious organ damage. Scleroderma patients may present hand dysfunctions that cause difficulties in performing daily activities. Hand dysfunction in scleroderma cases may be caused by RP (Raynaud phenomenon) with finger ulcers,^1^ arthralgia, arthritis, tendonitis,^1^ weakness^2^ and skin thickening, which cause loss of dexterity.^1,2^ Nacci et al.^1^ observed that the most limiting determinant was joint involvement. Others^3^ observed that rigidity, RP and weakness were the major contributors.

We studied hand dysfunction in 46 patients with scleroderma (four males and 42 females; mean age of 50.5 years; mean disease duration of 7.4 years). Twenty-nine presented the limited form, 11 presented the diffuse form and six cases overlapped with other connective tissue diseases. In this sample, 28.2% were employed workers; 10.8% were homemakers; 36.9% had retired due to health problems; and 15.2% had retired because of age. The patients were asked about the presence of hand symptoms and answered the Dreiser index questionnaire.^4^ This questionnaire contains 11 questions about activities of daily living. It is measured through a Likert scale (0 = no difficulty; 1 = slight difficulty; 2 = moderate difficulty; 3 = impossible to perform) and ranges from 0 (no dysfunction) to 33 (maximum dysfunction). It was initially created to evaluate hand osteoarthritis but has also been used in scleroderma cases.^1^ Patients with pain and stiffness were asked to grade their symptoms through a visual analogue scale (VAS) on which 0 corresponded to no symptoms and 10 to the maximum symptoms.

We found the following: RP in 93.5%; arthritis (present or previous) in 73.9%; hand stiffness in 73.9%; pain in the hand (overall) in 71.73%; arthralgia in 65.2%; skin ulcerations (present or previous) in 58.6%; and calcinosis in 26.6%. The Dreiser index results ranged from 0 to 25 (mean: 8.69 ± 8.02). The association between the Dreiser index and hand symptoms is shown in Table 1.

**Table 1:** Associations between Dreiser index and presence of hand signs and symptoms and autoantibody profile for scleroderma

	With the symptom(mean Dreiser ± SD)	Without the symptom (mean Dreiser ± SD)	P
Raynaud	9.0 ± 8.1	4.3 ± 5.1	0.35
Ulcerations	9.6 ± 8.14	7.3 ± 7.8	0.35
Arthralgia	11.8 ± 7.8	2.1 ± 2.7	< 0.0001
Arthritis	9.9 ± 8.0	6.3 ± 7.6	0.15
Calcinosis	10.5 ± 9.3	8.0 ± 7.5	0.34
Stiffness	10.2 ± 7.3	4.3 ± 8.6	0.003
Pain in the hand	11.5 ± 7.6	1.3 ± 2.1	< 0.0001

SD = standard deviation.

The mean VAS obtained for pain in the hand was 7.12 ± 2.26 and for stiffness, it was 6.17 ± 2.05. There were positive correlations between the Dreiser index values and the VAS for pain (r = 0.47; P = 0.006) and between the index and the VAS for stiffness (r = 0.69; P < 0.0001).

Hand dysfunction is not always taken into account in scleroderma cases, but Roberts-Thomson et al.^5^ showed that it can be as incapacitating as in rheumatoid arthritis.

We found that pain and stiffness were the symptoms that most affected functionality. Every effort should be directed towards treating these symptoms, in order to improve scleroderma patients’ wellbeing.

## References

[1] Nacci F, Righi A, Conforti ML (2007). Intravenous immunoglobulins improve the function and ameliorate joint involvement in systemic sclerosis: a pilot study. Ann Rheum Dis.

[2] Sandqvist G, Eklund M (2000). Hand Mobility in Scleroderma (HAMIS) test: the reliability of a novel hand function test. Arthritis Care Res.

[3] Malcarne VL, Hansdottir I, McKinney A (2007). Medical signs and symptoms associated with disability, pain, and psychosocial adjustment in systemic sclerosis. J Rheumatol.

[4] Dreiser RL, Maheu E, Guillou GB, Caspard H, Grouin JM (1995). Validation of an algofunctional index for osteoarthritis of the hand. Rev Rhum Engl Ed.

[5] Roberts-Thomson AJ, Massy-Westropp N, Smith MD (2006). The use of the hand anatomic index to assess deformity and impaired function in systemic sclerosis. Rheumatol Int.

